# Mapping the Proximity Interaction Network of STIM1 Reveals New Mechanisms of Cytoskeletal Regulation

**DOI:** 10.3390/cells10102701

**Published:** 2021-10-09

**Authors:** Jesse Gammons, Janith Halpage, Salvatore Mancarella

**Affiliations:** Health Sciences Center, Department of Physiology, University of Tennessee, Memphis, TN 38163, USA; jgammon5@uthsc.edu (J.G.); jhewahal@uthsc.edu (J.H.)

**Keywords:** cytoskeletal remodeling, STIM1, calcium, interactome, BioID, gelsolin

## Abstract

Stromal interaction molecule 1 (STIM1) resides primarily in the sarco/endoplasmic reticulum, where it senses intraluminal Ca^2+^ levels and activates Orai channels on the plasma membrane to initiate Ca^2+^ influx. We have previously shown that STIM1 is involved in the dynamic remodeling of the actin cytoskeleton. However, the downstream effectors of STIM1 that lead to cytoskeletal remodeling are not known. The proximity-labeling technique (BioID) can capture weak and transient protein-protein interactions, including proteins that reside in the close vicinity of the bait, but that may not be direct binders. Hence, in the present study, we investigated the STIM1 interactome using the BioID technique. A promiscuous biotin ligase was fused to the cytoplasmic C-terminus of STIM1 and was stably expressed in a mouse embryonic fibroblast (MEF) cell line. Screening of biotinylated proteins identified several high confidence targets. Here, we report Gelsolin (GSN) as a new member of the STIM1 interactome. GSN is a Ca^2+^-dependent actin-severing protein that promotes actin filament assembly and disassembly. Results were validated using knockdown approaches and immunostaining. We tested our results in neonatal cardiomyocytes where STIM1 overexpression induced altered actin dynamics and cytoskeletal instability. This is the first time that BioID assay was used to investigate the STIM1 interactome. Our work highlights the role of STIM1/GSN in the structure and function of the cytoskeleton.

## 1. Introduction

Stromal interaction molecules 1 and 2 (STIM1 and STIM2) are type 1 transmembrane proteins localized in the endoplasmic reticulum (ER), and the plasma membrane (PM) [1]. They contain an ER-luminal domain specialized for Ca^2+^ sensing and a cytoplasmic domain capable of regulated interaction with plasma membrane Orai channels [1,2]. STIM1 and STIM2 share high amino acid sequence homology and both isoforms can mediate Ca^2+^ influx by activating Orai channels gating after depletion of intracellular Ca^2+^ stores. The STIM-Orai complex is also known as store-operated Ca^2+^ channels (SOCCs), which produce store-operated Ca^2+^ entry (SOCE). Numerous studies have demonstrated that SOCCs play an essential role in various Ca^2+^ signaling pathways [3,4,5]. Although STIM1 is best known for forming signaling homodimers, it is now clear that STIM1 can engage in many lateral interactions with partners from diverse classes of proteins (interactome) for efficient transduction of Ca^2+^ changes across the plasma membrane [4,5].

Several members of the STIM1 interactome have been identified using a variety of approaches. Affinity purification of Orai1 in Jurkat T cells identified a partner of STIM1 (POST). The STIM1-POST complex binds to the P-type ATPase and the plasma membrane Ca^2+^ ATPases (SERCAs and PMCAs, respectively), Na/K-ATPase, importins-β, and exportins [6]. A functional expression screening with RNAi-mediated silencing led to the discovery of a negative SOCE-associated regulatory factor (SARAF) [7]. Glutathione S-transferase (GST) pull-down assays identified end-binding proteins (EB-1 and EB-3), members of the microtubule plus-end tracking proteins, as potential new partners of STIM1 [8].

Most of these techniques identified only strong, stable interactions that withstand harsh cell lysis conditions. However, weak and transient interactions between STIM1 and other partners in living cells escape detection. The engineered ascorbate peroxidase2 (APEX2) approach is more compatible with a live cell environment. Using this technique, Jing et al. discovered STIMATE, a protein that functions as a positive regulator of SOCE and affects STIM1 localization in ER-PM junctions. [9] APEX2 provides only a snapshot of proteins associated with STIM/Orai over a very short period of time (several minutes), not allowing the identification of STIM1 interactions that are involved in processes such as cell proliferation and migration that extend over a longer period of time.

Proximity-dependent biotin identification (BioID), is an innovative approach that uses a promiscuous biotin ligase attached to a target of interest and catalyzes the biotinylation of proteins residing in the vicinity of the target [10]. We generated a fusion protein joining BioID-HA and STIM1 and obtained the STIM1-BioID construct to generate a history of protein-protein associations occurring within a discrete subcellular domain during the labeling period. Biotinylated proteins were subsequently isolated, analyzed via mass spectrometry, and investigated as candidate interactors to further explore novel STIM1/SOCE functions.

We identified several proteins that reside in proximity to STIM1, among which there were known interactors of STIM1. These included integral proteins of the ER as well as components of the cytoskeleton. Gelsolin (GSN) was a novel protein revealed by STIM1-BioID. We demonstrate that STIM1 and GSN mediate cytoskeletal remodeling in fibroblasts and neonatal rat cardiac myocytes (NRCM). Our results show the unique role of STIM1 in controlling cytoskeletal remodeling and its potential involvement in the cellular and molecular pathways of mechanotransduction.

## 2. Materials and Methods

### 2.1. Molecular Cloning and Generation of Stable Cell Lines

For all experiments, stable and transiently transfected cell lines were maintained at 37 °C with 5% CO2 in Dulbecco’s Modified Eagle Medium (DMEM) supplemented with 10% fetal bovine serum (FBS) (Thermo Fisher Scientific, Waltham, MA, USA) and 1% Penicillin/Streptomycin (Pen-Strep, Corning Inc., Corning, NY, USA). Stable cell lines were generated by transfection with Lipofectamine 2000 (Thermo Fisher Scientific, Waltham, MA, USA). After recovery from transfection, cells were grown in DMEM containing 10% FBS, 1% PenStrep, and selection antibiotic, puromycin, 2 µg/mL, ensuring an effective positive selection of cells. Colonies were isolated, expanded, and screened for expression of the fusion proteins by Western blotting with anti-STIM1 and anti-HA flag antibodies (Cat. A8592, Millipore Sigma, St. Louis, MO, USA); see ‘Western blot analysis and antibodies’ below for details). Individual clones were selected to obtain cells with similar expression levels of the fusion protein. Cell area was measure in phase-contrast images manually outlined (ImageJ). To initiate a BioID experiment, low passage cells were plated at 20% confluence in 15 cm dishes as four replicates, with each replicate consisting of three 15 cm plates. After 24 h, biotin was added to the media to a final concentration of 10 µM and cells were incubated for an additional 16 h. After decanting the media, cells were dislodged from each plate by pipetting with ice-cold PBS. Cells were centrifuged at 1000× *g* for 5 min, and the PBS was decanted. Cells were washed once more with ice-cold PBS before proceeding to cell lysis. Cells were resuspended and lysed in 10 mL RIPA buffer (50 mM Tris-HCl, pH 8.0; 150 mM NaCl, 1% (*v*/*v*) NP-40, 0.5% (*w*/*v*) sodium deoxycholate, 0.1% (*w*/*v*) SDS, 1 mM DTT, and protease inhibitors (Protease Inhibitor Cocktail) by gentle rocking for 5–10 min at 4 °C. The cell lysate was clarified via centrifugation at 13,000 rpm for 30 min at 4 °C. The clarified lysate was retrieved and dialyzed twice against dialysis buffer (50 mM Tris-HCl, pH 8.0; 150 mM NaCl, 1 mM DTT, 0.01% Triton X-100) for 2 h per exchange. The dialysate was retrieved, supplemented with fresh protease inhibitors, and combined with 1 mL streptavidin-conjugated beads (Dynabeads MyOne Streptavidin T1, (Thermo Fisher Scientific, Waltham, MA, USA), and incubated for 1 h at 4 °C with gentle rocking. Bead/lysate mixtures were collected on a magnetic stand into a single 2 mL round-bottom microcentrifuge tube. The beads were washed four times with 2 mL RIPA buffer, with immobilization and solution removal performed on a magnetic stand. All plasmids used in this study were obtained from Addgene (MCS-BioID2-HA STIM1-BioID; Cat. 74224, https://www.addgene.org, accessed on 1 April 2021) and were generated by PCR subcloning (Epoch Life Science, Inc., Sugar Land, TX, USA).

### 2.2. Neonatal Cardiomyocytes Isolation

NRCM were isolated from hearts of 1–3-day-old Sprague-Dawley rats as described earlier [11,12]. Hearts were removed, atria were trimmed off, the remaining ventricles were minced, and the myocytes were dissociated with a Ca^2+^/bicarbonate free Hanks buffer (pH 7.5) containing 1% Pen-Strep, gentamicin (50 mg/L), heparin (5000 USP units/mL), DNase (2 mg/mL), and trypsin (1.5 mg/mL) at 25 °C with gentle shaking. The dissociated NRCM were pre-plated for 2 h to reduce non-myocyte contamination; the medium was then replaced with DMEM (GIBCO, Carlsbad, CA, USA) supplemented with 5% FBS (Hyclone Laboratories, Logan, UT, USA), Bromodeoxyuridine / 5-bromo-2′-deoxyuridine (BrdU) (0.1 mM; Millipore Sigma, St. Louis, MO, USA) vitamin B_12_ (1.5 mM; Millipore Sigma, St. Louis, MO, USA), and antibiotics. After the first 24 h, the medium was replaced again with DMEM supplemented with: insulin (10 µg/mL; Millipore Sigma, St. Louis, MO, USA), transferrin (10 µg/mL; Millipore Sigma, St. Louis, MO, USA), vitamin B_12_ (1.5 mM; Millipore Sigma, St. Louis, MO, USA), and antibiotics. NRCM were grown on fibronectin-coated coverslips. Cells were transfected with STIM1-YFP, Scrambled GSN shRNA, GSN shRNA using lipofectamine 3000 (Life technologies). Live-cell imaging was conducted using the IX83 Olympus microscope attached to the Zyla 5.5 sCMOS camera. 

### 2.3. Mass Spectrometry

The biotinylated multi-protein complex (MPC)-specific proteins of interest comprises background proteins, including endogenous biotinylated proteins and proteins non-specifically bound to the beads. To discriminate the MPC specific proteins from the background proteins, control analysis was performed in parallel; under the control conditions, the protein of interest is expressed without a fused enzyme, and, thus, only background proteins are isolated/analyzed. Experimental and control samples derived from equal amounts of beads were processed (on-beads reduction/alkylation, Lys-C/trypsin digestion) and 10% of a processed sample was analyzed for peptide/protein identification and label-free quantification (LFQ) using mass spectrometry-based LC/MS/MS analysis. An estimated 2 µg of protein was processed and 0.2 µg of digested protein was analyzed per sample under the experimental conditions. For statistical validation of results, four biological replicates were analyzed per experimental and control conditions. LC/MS/MS analysis with 100 min LC gradient was performed on an Orbitrap Fusion Lumos mass spectrometer operating in line with an UltiMate 3000RSLCnano UHPLS system (Thermo Fisher, Waltham, MA, USA). Post-acquisition analysis of raw MS data for peptide/protein identification and quantification was performed within a mass informatics platform Proteome Discoverer 2.2 (Thermo Fisher, Waltham, MA, USA) using Sequest HT search algorithm and mouse protein database (SwissProt, Mus musculus, TaxID 10090, v.2017-10-25, 25,097 entries). The abundance (Area Under Curve) of each identified peptide and corresponding parent protein was determined in each analyzed sample. The average abundance of each identified/quantified protein was determined per experimental and control group of samples; corresponding CVs and the relative abundance of each protein (molar ratio, experiment vs. control) were calculated. Identification and quantification of peptides/proteins were validated at 0.01 False Discovery Rate (FDR), and p-value of 0.05 corrected for multiple testing, respectively. Unlike the background proteins, each protein specific to the MPC of interest was expected to be more abundant in the experimental group of samples than in the control group and to show relative abundance significantly exceeding 1. The statistically validated proteins with relative abundances exceeding 2-fold were selected as candidate MPC proteins associated with the protein of interest; the candidate proteins were further validated based on functional assays, Western blot analyses, and immunohistochemistry.

### 2.4. Immunoblots and Immunofluorescence

Lysates and eluates were run on 4–12% polyacrylamide gels (NuPage, Thermo Fisher Scientific, Waltham, MA, USA) and transferred onto PVDF (Immobilon-FL, Millipore Sigma, St. Louis, MO, USA) for 1.5 h at 300 mA constant current. Blots were blocked for 10 min with TBST + 5% dry milk (*w*/*v*), and immunoblotted with appropriate antibodies. All antibodies were diluted in TBST +5% milk (*w*/*v*). Primary antibodies were incubated overnight at 4 °C, while secondary antibodies were incubated for 1 h at room temperature. Antibodies used in this study were Anti-alpha smooth muscle actin (SMA) (Cat. 5694, Abcam, Eugen, OR, USA,), anti-HA-Tag (Cat. 3724, Cell Signaling), anti-vinculin (Cat. ab18058, Abcam, Eugen, OR, USA), rhodamine-phalloidin (Cat. R415, Thermo Fisher, Waltham, MA, USA), anti-tubulin (Cat. 66240, Proteintech, Rosemont, IL, USA).

For immunofluorescent experiments, MEF/NRCM were cultured on coverslips to a density of 5 × 10^4^ cells/coverslip. After 5 days at 37 °C, cells were then fixed, permeabilized, and stained for 90 min at room temperature with primary antibodies overnight at 4 °C. Secondary antibodies and/or rhodamine-phalloidin for 90 min at room temperature. Coverslips were then washed with PBS and mounted on slides for imaging using a Zeiss 710 confocal microscope. Quantification of immunofluorescence was obtained by quantifying skeletonized images taken with the same laser intensity in ImageJ.

### 2.5. Determination of F-actin/G-actin Ratio

The F/G actin ratio was determined using the G-actin/F-actin Kit according to the manufacturer’s instructions (Cytoskeleton Inc., Denver, CO, USA). Briefly, cells were collected and lysed in F-actin stabilizing lysis buffer (Cytoskeleton Inc., Denver, CO, USA). After removal of debris by a short centrifugation at 350× *g*, lysates were subjected to ultracentrifugation (100,000× *g*) for 1 h to separate F-actin (pellet) from soluble G-actin (supernatant). An equal amount (as used in the lysis step) of F-actin depolymerization buffer was added to the pellet containing F-actin. An equal amount of both suspensions was loaded onto the gel and analyzed using an Odyssey Infrared Imaging System (Li-Cor Bioscience, Lincoln, NE, USA).

### 2.6. Data Analysis

All data were expressed as mean ± SEM. Comparisons between the control and experimental groups were performed with the Student’s *t*-test. Comparisons between > two groups were conducted on raw data with ANOVA followed by Bonferroni correction, which was calculated with GraphPad Prism, version 9 (GraphPad Software; San Diego, CA, USA). Experiments using isolated cardiomyocyte (*n*) were obtained from several hearts (N) preparations.

## 3. Results

### 3.1. Identification of STIM1 Interactome

**Figure 1:**
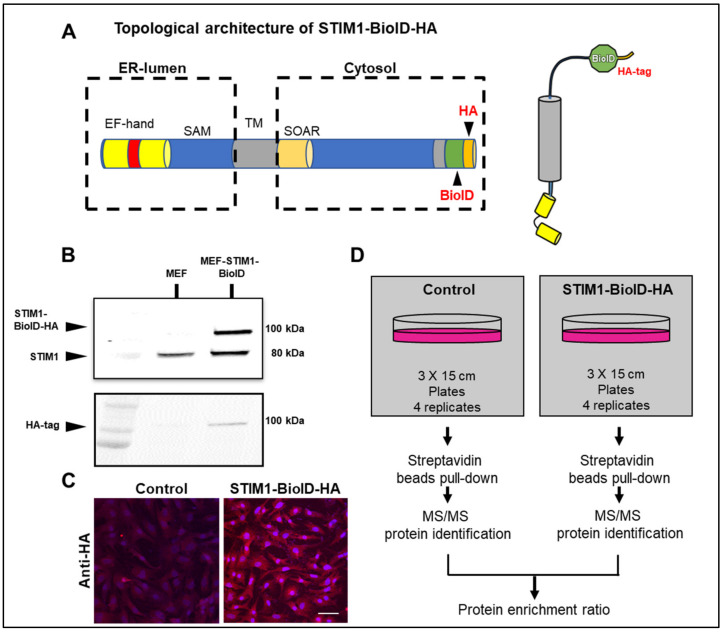
Generation of STIM1-BioID construct and BioID assay workflow. (**A**) Schematic representation of the mammalian STIM1 protein with color-coded domains, BioID- and HA-tag were positioned at the STIM1 C-terminus. (**B**) Expression of STIM1 and STIM1-BioID was validated with Western blot and antibodies against STIM1 and Anti-HA-tag. (**C**) Confocal images of immunofluorescence staining of fixed control and MEF-STIM1-BioID. Cells were stained with DAPI and anti-HA-tag, pictures were taken at 405 nm and 564 nm. Scale bar: 20 μm. (**D**) Biotinylation and mass spectrometry (MS) analysis workflow: cultured control and MEF- STIM1-BioID were incubated with biotin provided in the media for 16 h and then lysed under stringent conditions. Biotinylated proteins were collected on streptavidin-conjugated beads for subsequent analysis and identification.

To perform unbiased quantitative profiling of the STIM1 interactome, we generated a human STIM1 C-terminally tagged with BioID construct (STIM1-BioID). The BioID portion was fused to a hemagglutinin (HA)-tag (Figure 1A). In the presence of biotin, BioID covalently tags nearby endogenous proteins on lysine residues with a labeling radius estimated to be around 10 nm depending on the local environment [13]. Next, we generated MEF stably expressing STIM1-BioID. Analysis of immunoblots from cell lysates revealed successful detection of endogenous STIM1 in control MEF, while both endogenous STIM1 and exogenous STIM1-BioID were detected in MEF-STIM1-BioID (Figure 1B). The presence of STIM1-BioID in stable transfected MEF was further confirmed by an anti-HA-tag antibody (Figure 1B). Anti-HA antibody showed correct intracellular localization and uniform protein expression of STIM1-BioID (Figure 1C). After incubation with biotin, the STIM1 interactome was obtained by streptavidin pull-down followed by quantitative mass spectrometry analysis. All BioID experiments with tagged STIM1 were performed in quadruplicate using a label-free quantitative proteomic approach. Controls with non-transfected MEF incubated with biotin were also included (Figure 1D).

**Figure 2:**
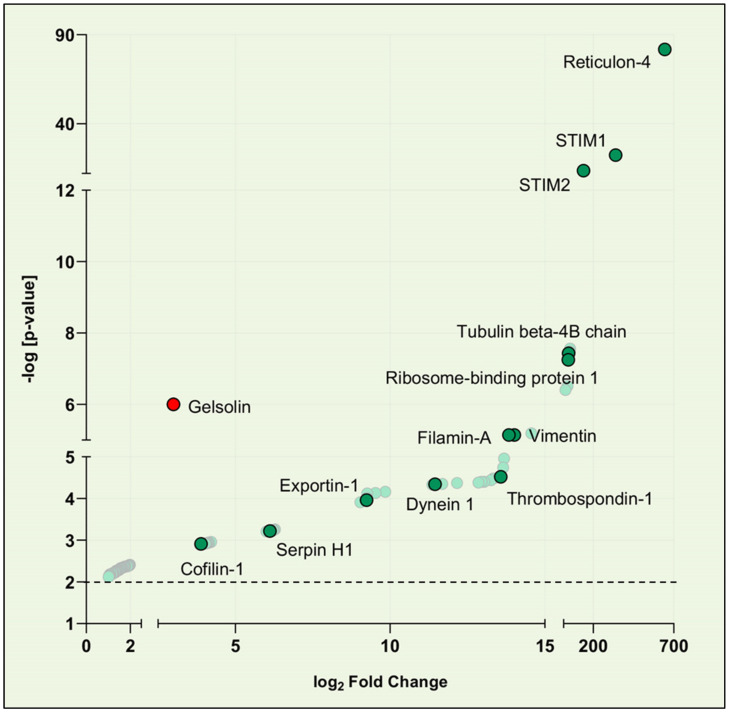
A volcano plot depicting quantified STIM1 proteome identified by MS. For clarity, only the most aboundant proteins (−log[*p*-value] > 2.0) are shown, known STIM1 interactors are labeled. The *x*-axis shows the average fold change (log2) in protein abundance compared to control, and the *y*-axis shows the average adjusted −log[*p*-value].

Most of the biotinylated proteins identified were known to reside in the cytoplasm and the ER membrane (Figure 2). The most abundant biotinylated protein identified was Reticulon 4 (RTN4). Proteins of the RTN family localize to the ER and are responsible for sculpting ER elements, RTN were reported to be necessary for SOCE function and regulation [14,15,16,17]. STIM1 and STIM2 were abundantly biotinylated due to their capability to oligomerize. Interestingly, our data suggest that STIM2 comes in close proximity to STIM1 and may cooperate in the regulation of long-term cellular processes. We detected two nuclear protein-transporters, exportin1 and transportin1, previously reported to interact with Orai1 [6,18]. Vimentin and filamin-A, which were previously reported to “facilitate” the SOCE-dependent signaling pathway by optimizing the localization of STIM1, were also biotinylated in our assay. Thrombospondin-1, previously reported to bind STIM1 [19], was biotinylated as well. Cofilin-1 was also picked up in our screeningis, cofilins are essential in actin regulation and are involved in SOCE regulation. Overall, the presence of known “baits” in the pool of biotinylated proteins further validated our approach.

Among the biotinylated proteins that associate and/or are proximate to STIM1, GSN was identified as a novel potential STIM1 partner. GSN is a potent member of the actin-severing GSN/villin superfamily, as it severs with nearly 100% efficiency and caps the actin fast-growing “barbed” end, thus favoring depolymerization from the pointed end [20]. GSN is present in most animal cells, and its severing activity is Ca^2+^ and polyphosphoinositide-4,5-bisphosphate-dependent [21]. At sub-micromolar Ca^2+^ concentrations, Ca^2+^-regulated capping and severing activities can occur independently of one another [22]. We focused on further validating GSN as part of the STIM1 interactome for several reasons. First, GSN is implicated in many relevant cellular processes, including cytoskeletal remodeling during heart development, pathological cardiac remodeling, and some forms of cancer [23,24]. Second, to our knowledge, a functional connection between STIM1 and GSN has not been reported previously. Third, placing GSN near STIM1 may finally elucidate the role of STIM1 and SOCE in cytoskeletal remodeling and mechanotransduction.

### 3.2. STIM1 Overexpression Alters Cell Size and Perturbs the Cytoskeleton Network

**Figure 3:**
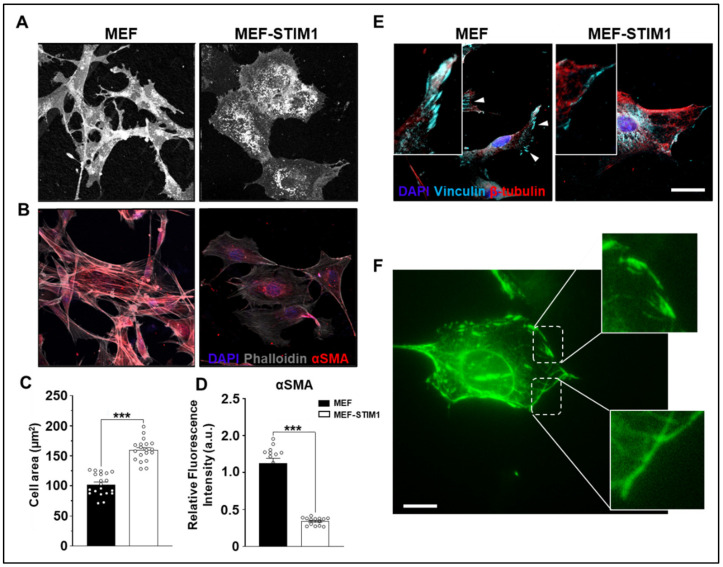
Morphology and myofilament distribution in STIM1-BioID MEF. (**A**) Phase-contrast images of live control and STIM1-BioID MEF were acquired five days after plating. Note the elongated morphology of most control cells, in contrast to cells expressing STIM1-BioID, which appeared larger and with fewer protrusions. (**B**) Actin distribution in control and STIM1-BioID MEF. Cells were visualized after plating using Alexa-phalloidin to stain F-actin (pseudo-colored gray) and an anti-α-actin (pseudo-colored red) and DAPI (blue). (**C**) Cell area quantification analysis. (**D**) Relative 488-α-actin fluorescence quantification analysis. (**E**) Fluorescence micrographs of cells stained with Anti-tubulin, anti-vinculin (indicated by arrows), and DAPI. (**F**) Live-cell imaging of a MEF expressing STIM1-YFP. Arbitrary units (a.u.). Bar = 50 μm. Error bar = SD, *** *p* < 0.001.

MEF expressing STIM1-BioID exhibited a larger cell area and spread size when compared to control MEF (Figure 3A,C). Similar observations of cells size increase were made from myoblast carrying a STIM1 gain-of-function mutation [25]. Since the BioID screening assay revealed that GSN is in the vicinity of STIM1, we tested the hypothesis that STIM1 overexpression alters cell cytoskeleton and actin distribution. Indeed, cells stained with phalloidin revealed abundant stress fibers with elongated spindle shapes in MEF controls but were seen to a lesser extent in STIM1-BioID expressing MEF (Figure 3B,D). Therefore, STIM1 overexpression was sufficient to reduce the presence of actin stress fibers.

To characterize the effects of STIM1 overexpression on cell protrusions and distribution in the cell periphery, we studied the distribution of vinculin, a highly conserved focal adhesion protein. Vinculin was localized in stable, well-defined structures resembling the focal adhesions in control cells, whereas staining intensity was reduced and more diffused in MEF-STIM1-BioID (Figure 3E). The distribution of vinculin is consistent with its role in focal adhesions formation and maintenance, as well as in the process of cell spreading. Therefore, these results suggest STIM1 expression level regulates focal adhesions stability and distribution.

STIM1-YFP overexpression recapitulates the cellular phenotype of STIM-BioID, showing STIM1 localization in a filamentous-like shape at the cell periphery and in structures resembling focal adhesion (Figure 3F) and further implicating STIM1 in cytoskeletal remodeling.

**Figure 4:**
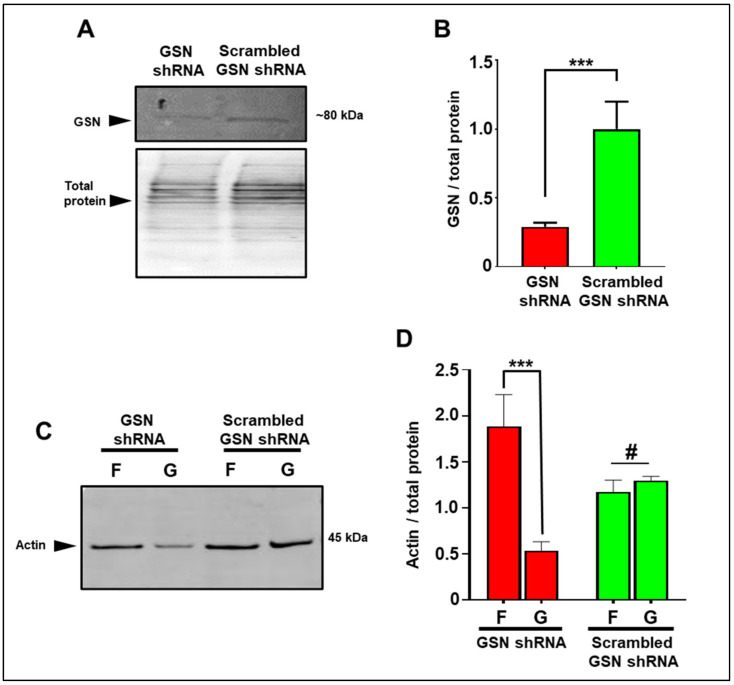
Relationship between GSN and cytoskeletal remodeling. (**A,B**) Efficiency of GSN shRNA knockdown vs. scrambled GSN shRNA in MEF-STIM1-BioID. (**B**) Barr graph quantification of *GSN* knockdown. (**C**) Reorganization of actin, F-actin—F and G-actin—G in MEF-STIM1-BioID transfected with GSN targeting shRNA or scrambled shRNA. (**D**) Quantification analysis of G-actin and F-actin. *GSN* knockdown resulted in a potent accumulation of F-actin. Error bar = SD, # *p* > 0.05, *** *p* < 0.001.

To gain direct evidence that STIM1 controls actin remodeling through GSN, we knocked down *GSN* expression in MEF expressing STIM1-BioID using short hairpin RNA (shRNA). Targeted GSN shRNA resulted in about 80% reduction of GSN protein levels compared to cells expressing scrambled GSN shRNA (Figure 4A,B).

Next, we investigated the effects of GSN downregulation on the distribution of F-actin (filamentous actin) and monomeric G-actin (globular actin) in STIM1 overexpressing MEF. Silencing GSN showed increased presence of F-actin and a decreased G-actin. In contrast, cells transfected with scrambled GSN shRNA exhibited an equal amount of F-actin and G-actin (Figure 4C,D). These results indicated that high levels of STIM1 expression activate GSN to sever actin, further demonstrating that GSN is downstream to STIM1 and mediates actin depolymerization and cytoskeletal remodeling.

### 3.3. STIM1 and GSN Actively Remodel Actin in Neonatal Cardiomyocytes

GSN and STIM1 proteins are present in murine and human hearts, and their expression levels are significantly upregulated during cardiac development [12,26,27]. Much experimental evidence indicates a crucial role of GSN-mediated actin remodeling during heart remodeling in the pathogenesis of heart failure [24].

**Figure 5:**
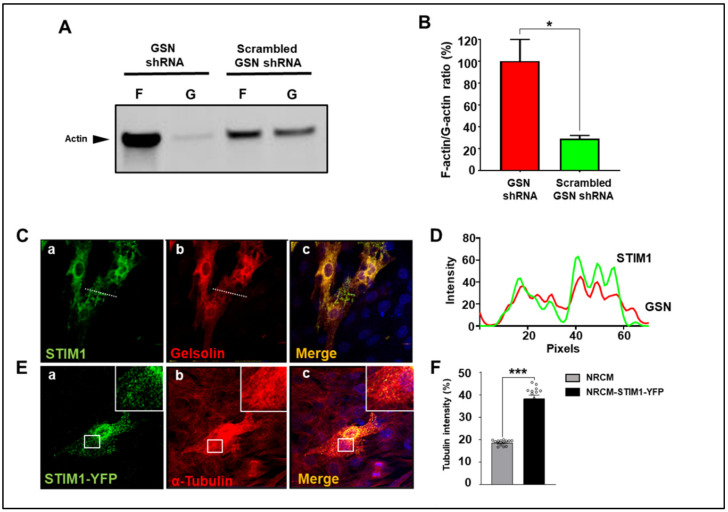
STIM1 induces cytoskeletal remodeling in NRCM. (**A**) Blot showing F- and G- actin in the STIM1 overexpressing NRCM. *GSN* knockdown cells there is accumulation of F-actin, in contrast to cells transfected with scrambled GSN shRNA. (**B**) Quantification analysis of F/G actin ratios. (**C**) NRCM transiently overexpressing STIM1-YFP (a) and co-stained with anti-GSN antibody (b) Merging the fluorescence signal reveals colocalization between STIM1 and GSN (c). (**D**) Line-scan profiles of fluorescence intensity for NRCM (form **C**, a and b). The green line indicates STIM1 spatial distribution, and the red line indicates GSN distribution. (**E**) Micrographs of NRCM transiently expressing STIM1-YFP (a) and stained with anti-tubulin antibody (b), merged channels (c). (**F**) Tubulin quantification by fluorescence intensity between NRCM and NRCM expressing STIM1-YFP. Data are from three independent experiments, * *p* < 0.05, *** *p* < 0.001.

Given that our interactome analysis uncovered a functional relationship between STIM1 and GSN, we investigated whether STIM1/GSN machinery mediates actin filament organization in NRCM. These cells express high STIM1 and GSN, probably because they are undergoing intense cytoskeletal remodeling. We have reported that STIM1 overexpression negatively impacts the actin network in these cells [12]. We found that GSN downregulation in NRCM overexpressing STIM1 resulted in abundant F-actin and small amounts of G-actin. In contrast, STIM1 overexpression in cells transfected with scrambled GSN shRNA induced a decrease of F-actin and an increase in G-actin, further suggesting that GSN is downstream to STIM1 (Figure 5A,B).

Furthermore, we observed that STIM1-YFP colocalized with GSN in NRCM (Figure 5C). These observations were more qualitatively confirmed by the line scanning profiles of fluorescent intensity of the selected NRCM (Figure 5D). Immunocytochemical analysis of cardiomyocytes overexpressing STIM1 showed a sharp increase of tubulin density when compared with control NRCM (Figure 5E,F). Combined, these data suggest that, in NRCM, STIM1 and GSN mediated cytoskeletal remodeling and that GSN activation could induce weakening of the actin cytoskeleton triggering compensatory responses such as upregulation of other cytoskeletal components.

## 4. Discussion

We applied proximity-dependent biotin identification (BioID) to map the STIM1 interactome network. GSN was identified as a new member of STIM1 interactome. We show that STIM1 and GSN orchestrate cytoskeletal remodeling in fibroblasts and neonatal cardiomyocytes.

By attaching BioID to the cytoplasmic portion of STIM1 we identified a number of proteins whose functions have been reported to be altered by SOCE or are involved in regulating STIM1 ER distribution and activation. We are now able to place these proteins within the STIM1 interactome and show that over time they come in close proximity to STIM1 and can influence, or be influenced, by both STIM1 and SOCE. It is intriguing that STIM1 and STIM2 were abundantly biotinylated in the absence of external stimuli or artificial store emptying. This suggests that STIM isoforms oligomerize in spatially restricted microdomains to mediate critical cellular functions.

**Figure 6:**
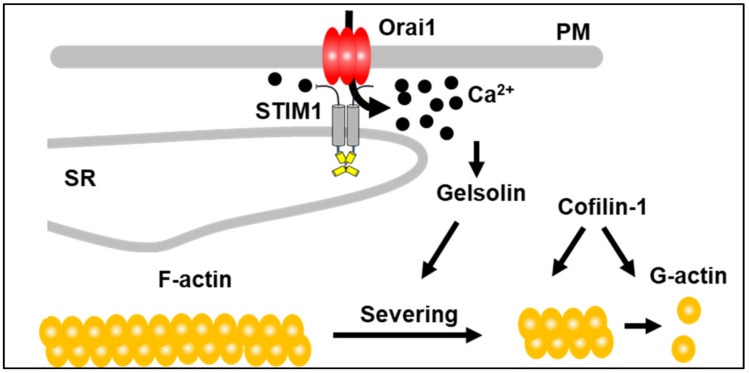
Regulation of cytoskeleton density by STIM1. Explanation in the text.

Several studies, including work from our laboratory [12,28], have implicated STIM1 and SOCE in the regulation of smooth muscle cytoskeleton during cell migration, cardiac remodeling and the formation of the immunological synapse [12,28,29]. However, an important remaining question is how STIM1-dependent signaling integrates local Ca^2+^ microdomains to activate cytoskeletal remodeling. GSN is a Ca^2+^ binding protein that nucleates actin filament assembly, blocks the fast-exchanging ends of actin filaments, and shortens actin filaments. GSN severing activity requires supraphysiological intracellular Ca^2+^ concentrations (in the order of 10 µM) and is inhibited following the removal of Ca^2+^ ions. Interestingly, a bulk increase of cytoplasmic Ca^2+^ fails to induce GSN activation [30]. Since a significant fraction of GSN is localized at the plasma membrane [31], it was postulated that areas near the Ca^2+^ selective ion channels pore might reach higher Ca^2+^ concentrations than the bulk phase. Several ion channels were thought to mediate the local Ca^2+^ entry required to activate GSN. However, their identity has remained elusive. Our study places GSN near STIM1, which is suggestive of SOCE being the source of the Ca^2+^ responsible for activating GSN. Indeed, STIM1 can trigger the generation of local Ca^2+^ microdomains by activating Orai channels, as the Ca^2+^ concentration near the mouth of the channels is estimated to reach concentrations between 6 and 12 µm [32], a range that can activate GSN and initiate precisely-controlled submembrane actin remodeling, a common early event in cell activation and locomotion [30]. Importantly, our screening indicated that cofilin-1 was among the biotinylated targets. Cofilins accelerates actin dynamics by severing and disassembling actin filaments. It is compelling that cofilin-1 has been shown to cooperate with GSN to sever actin filaments and plays a role in the activation of SOCE [33,34]. Based on our findings, we proposed a model where STIM1, GSN, and other players like cofilin1 regulate acting filament polymerization/depolimerization (Figure 6). 

Overall, many positive hits produced by our BioID approach were proteins involved in the cytoskeletal stability and establish STIM1 as a critical player in cytoskeletal dynamics. Our results show that STIM1 expression alters focal adhesion stability and morphology supporting previous findings showing that STIM1 knockdown increases focal adhesions lifetime [35]. Furthermore, it was shown that GSN colocalizes with vinculin and actin, orchestrating cell migration and podosome formation in the colon adenocarcinoma cell line [35,36]. The STIM1-dependent calcium signaling that regulates GSN and cofilin1 to orchestrate filament remodeling deserves further investigation. 

It is clear that the influence of STIM1 expression and activity on actin-regulatory molecules is a cell-type specific function. To carry out their functions, fibroblasts must migrate within the interstitial space, this requires locally restricted F-actin degradation to facilitate locomotion [37,38]. In contrast, in myocytes focal adhesions are less dynamic, and finely tuned Ca^2+^ signaling is essential for regulating important cell function and remodeling other that migration [39]. Nevertheless, our results suggest that STIM1 and GSN are vital components of focal adhesion dynamic.

Our discovery that GSN is part of the STIM1 interactome contributes to further understanding of the role of these proteins during development and pathological conditions. This is particularly interesting since both STIM1 and GSN have been independently implicated in critical pathological processes like the migration of metastatic cell lines and fibrosis [40,41,42,43]. Validation of our results in NRCM explains why these proteins are upregulated during heart development and contribute to cardiac heart failure progression, two stages characterized by intense cell and organ remodeling. We also showed that weakening of the actin cytoskeleton induced by STIM1 overexpression induces compensatory responses with upregulation of other proteins probably to maintain cytoskeletal integrity. These data support previous observations that partial loss of actin resulted in tubulin overexpression during pathological cardiac remodeling, leading to heart stiffness and decreased contractility contributing to the progression of heart failure [44].

In conclusion, STIM1 promotes cytoskeletal remodeling through activation of GSN and actin remodeling, and this could be a new target for therapeutical purposes to either accelerate or prevent remodeling during the disease state.

## Data Availability

The data presented in this study are available on request from the corresponding author.
